# Scale-Hybrid Group Distillation with Knowledge Disentangling for Continual Semantic Segmentation

**DOI:** 10.3390/s23187820

**Published:** 2023-09-12

**Authors:** Zichen Song, Xiaoliang Zhang, Zhaofeng Shi

**Affiliations:** School of Information and Communication Engineering, University of Electronic Science and Technology of China, Chengdu 611731, China; xlzhang@std.uestc.edu.cn (X.Z.); zfshi@std.uestc.edu.cn (Z.S.)

**Keywords:** continual semantic segmentation, knowledge distillation, scale-hybrid group semantic distillation

## Abstract

Continual semantic segmentation (CSS) aims to learn new tasks sequentially and extract object(s) and stuff represented by pixel-level maps of new categories while preserving the original segmentation capabilities even when the old class data is absent. Current CSS methods typically preserve the capacities of segmenting old classes via knowledge distillation, which encounters the limitations of insufficient utilization of the semantic knowledge, i.e., only distilling the last layer of the feature encoder, and the semantic shift of background caused by directly distilling the entire feature map of the decoder. In this paper, we propose a novel CCS method based on scale-hybrid distillation and knowledge disentangling to address these limitations. Firstly, we propose a scale-hybrid group semantic distillation (SGD) method for encoding, which transfers the multi-scale knowledge from the old model’s feature encoder with group pooling refinement to improve the stability of new models. Then, the knowledge disentangling distillation (KDD) method for decoding is proposed to distillate feature maps with the guidance of the old class regions and reduce incorrect guides from old models towards better plasticity. Extensive experiments are conducted on the Pascal VOC and ADE20K datasets. Competitive performance compared with other state-of-the-art methods demonstrates the effectiveness of our proposed method.

## 1. Introduction

Semantic segmentation [1,2] is a fundamental task in the field of computer vision that aims to assign a category to each pixel in an image. With the help of Convolutional Neural Networks (CNNs), semantic segmentation methods have achieved significant progress under the condition of all of the fixed classes that have been given. However, the main challenge in the real-world lies in the constantly changing environment (i.e., the new classes are generated progressively), which means a semantic segmentation model needs to continually learn from newly emerged classes while preserving the learned knowledge from old classes without retraining from scratch. Such a learning process is called Continual Semantic Segmentation (CSS), which has gained widespread interest from researchers and plays an important role in wide potential applications in dynamically changing environments, such as automatic driving, medical imaging, robotics, augmented reality, and so on.

A straightforward solution is to fine tune [3] the trained old model on data from the new class to adjust the parameters to fit the distribution of the new class without using the old class of data. Nevertheless, the model suffers from a catastrophic forgetting problem [4,5] when it is updated incrementally by a gradient-based update method, which will directly lead to rapid degradation of the model performance, i.e., the model will forget how to solve the old classes after learning the new classes due to the interference caused by the parameter updation. To alleviate the problem, some researchers have attempted to adopt the knowledge distillation strategy [6,7,8,9,10,11], which transfers knowledge from the old class to the new class of models to preserve the model’s original capacity. Despite the success of the knowledge distillation-based method for CSS, two common limitations remain. The first limitation is inadequate distillation. Specifically, only the knowledge of the last layer of the encoder is transferred by simply pooling the feature map, which fails to leverage the richer knowledge of the old model such as multi-scale object information, spatial position, and channel semantics. The second limitation is the semantic shift [12]: the old CSS models consider all data that is not the current class as background, which may contain non-background information in the new task. However, existing CSS methods force distill the entire feature map from the old task decoder to the new task, which may disturb the learning of new classes since some new classes are mistakenly considered as background by the old model.

To address the aforementioned problems, we propose a novel scale-hybrid group distillation with the knowledge disentangling continual semantic segmentation method. In particular, a scale-hybrid group distillation for encoding is proposed for transferring richer semantic knowledge from the feature encoder of the old model to avoid inadequate distillation. On the one hand, we perform multi-scale distillation of the encoder to preserve comprehensive semantic information. On the other hand, we design a group pooling strategy to retain the spatial position and channel semantic knowledge. In addition, we propose the knowledge disentangling distillation method composed of old class distillation and new class learning for decoding to reduce the semantic shift of the background class. Unlike previous indiscriminate distillation, we utilize the old model to generate pseudo labels for the current task, and then only focus on the knowledge transfer from the old non-background classes to avoid the semantic shift where the class is considered as background in the old task and foreground in the new task. Additionally, the new class learning is supervised by the cross entropy loss function in order to enhance the plasticity of the model.

The main contributions can be summarized as follows:

- We propose a scale-hybrid group distillation (SGD) for encoding to transfer richer semantic knowledge from the old model’s feature encoder in different scales in a novel group pooling manner to preserve comprehensive knowledge without the catastrophic forgetting problem.
- We propose a knowledge disentangling distillation (KDD) for decoding to decompose the learning of old and new knowledge based on the corresponding model. This approach can reduce the interference of incorrect guides from old models for the new knowledge.
- Extensive experiments on Pascal VOC and ADE20k datasets are conducted on the typical continual semantic segmentation settings, and the results demonstrate the effectiveness of our proposed method.

## 2. Related Work

In this section, we firstly make a overview of two important research fields: semantic segmentation and continual learning. Then, we further make an in-depth exploration of recent advancements in continual semantic segmentation field.

### 2.1. Semantic Segmentation

In recent years, significant progress has been made in the field of semantic segmentation benefit from the availability of large datasets [13,14,15,16] and advancements [1,2] in deep convolutional neural networks. Long et al. [1] proposed an end-to-end architecture for semantic segmentation called fully convolutional networks (FCN), which can output pixel-wise prediction of the object class. However, FCN suffers from the loss of spatial information and insufficient contextual information. Chen et al. [17,18,19] proposed the Deeplab series to capture more spatial information through atrous convolutions. Some works [2,20,21,22] have adopted an encoder-decoder structure for retaining spatial information. In order to capture contextual information in images, some works [23,24,25,26,27] have adopted an attention mechanism to build up connections between image contexts. Recently, semantic segmentation approaches based on the transformer architecture have had a great deal of success [28,29,30,31,32], mainly in their ability to capture long-range dependencies in images. Despite the remarkable achievement of the semantic segmentation field, these methods are not capable of dealing with the new emerging classes of continual learning, which are more applicable to real-world scenarios.

### 2.2. Continual Learning

Continual learning aims to adapt and learn from new data while retaining knowledge acquired from previous data. However, catastrophic forgetting of old knowledge is a major challenge when learning new knowledge. To address this issue, some approaches have been proposed for continual learning, including replay-based methods [33,34,35,36,37,38], regularization-based methods, and architecture-based methods. Replay-based methods solve the catastrophic forgetting problems by retaining the old knowledge in the learning of new data. The stored old knowledge can be divided into many types, including partial raw data [33,36], synthetic data [34,35], and prototype information [37,38]. Without the need of storing old data, regularization-based methods adopt regularization techniques to encourage the model to retain previous knowledge, including the knowledge distillation [39,40,41,42,43], adversarial learning [44,45], and vanilla regularization methods [46,47,48]. Besides, the architecture-based approaches [49,50,51] dynamically adjust the network architecture to preserve the learned knowledge from the old task while acquiring new information from the current task. Recently, continual learning techniques have been applied to a series of computer vision tasks, including object detection [52,53], semantic segmentation [6,7,12], and instance segmentation [54,55].

### 2.3. Continual Semantic Segmentation

Continual semantic segmentation (CSS) [6,7,11,12,56,57] is a very challenging task in computer vision, which aims to solve the catastrophic forgetting [46] in semantic segmentation. ILT [6] first proposed the continual semantic segmentation task and a Deeplab-based [18] CSS framework. Cermelli et al. [12] further built unbiased knowledge distillation to avoid catastrophic forgetting.

In recent years, more CSS methods [7,8,9,10,11,56,57,58] have been proposed. SDR [8] made full use of the prototype matching to reduce forgetting and improve the representation ability of new classes. PLOP [7] extracted multi-scale features in intermediate layers for knowledge distillation. Replay-based RECALL [9] hoped to add extra data of old classes and the background class from the online website or by using the GAN network. Taking into account the influence of class similarities, REMINDER [57] built different weights during the processing of distillation. Architectural-based RCN [10] creatively designed two parallel network branches for storing old knowledge and learning new categories to avoid forgetting problems. RBC [11] believed that the background of old classes in the new image is more similar to the old image, which can significantly exacerbate the old class forgetting and the new level of learning. So a biased-context-rectified CSS framework was proposed, which decoupled different classes by using context-rectified image-duplet learning.

However, the above existing methods directly utilize the knowledge of old models by merely distilling the last layer of the feature encoder and simply pooling the feature map for distillation without paying attention to the different impacts of backgrounds and old classes.

## 3. Method

**Figure 1:**
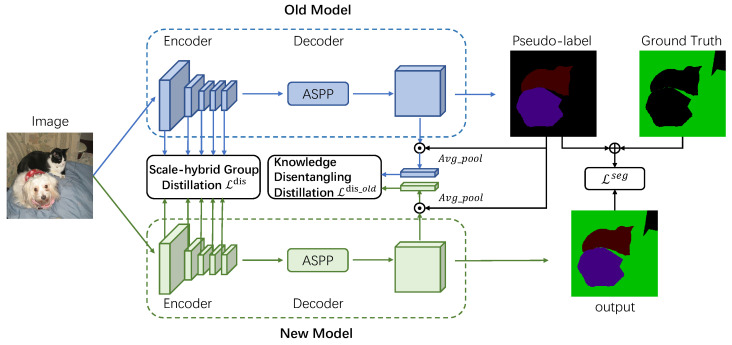
An overview of our method. Our method consists of the Scale-hybrid Group Distillation in the encoder and the Knowledge Disentangling Distillation in the decoder. For the encoder, we propose group distillation for feature layers with different scales to preserve the old knowledge more adequately. For the decoder, we distill the output feature map of Atrous Spatial Pyramid Pooling (ASPP) module. Specifically, we disentangle the old and new classes and only distill the old class features, so as to alleviate the catastrophic forgetting background caused by the semantic shift problem.

The overall framework of our method is shown in Figure 1. Given an image, we first input the image into the old model in the last step to predict the segmentation masks of the old classes learned in the previous step. Then, the predicted masks are used as pseudo-labels of the old classes, and they are combined with the ground truth of the new classes to train the current model. Next, to prevent catastrophic forgetting, we propose scale-hybrid group distillation (SGD) for the encoder and knowledge disentangling distillation (KDD) for the decoder. In the following sections, we first introduce the basic settings and preliminaries of continual semantic segmentation in Section 3.1. The framework of our method is then illustrated in Section 3.2. Moreover, we present the proposed scale-hybrid group distillation in Section 3.3 and knowledge disentangling distillation in Section 3.4.

### 3.1. Preliminaries

Compared with traditional semantic segmentation with all classes of labels available, the continual semantic segmentation (CSS) task divides the dataset into multiple subsets based on classes, each of which only contains a part of the class labels. The model learns one subset at each learning step without labels from previous subsets, and there is no class intersection between different subsets.

Given a multi-step dataset $D=\{D^{1},D^{2},\ldots ,D^{T}\}$, and the corresponding classes $C=\{C^{1},C^{2},\ldots ,C^{T}\}$ contained in each subset of D, where *T* is the total learning steps. The subset $D^{t}$ contains a series of sample pairs $\left\{I_{i}^{t},G_{i}^{t}\right\}$, where $I_{i}^{t}\in R^{3\times H\times W}$ is the input images and $G_{i}^{t}$ is the corresponding ground truth, *H* and *W* are the height and width of the input images, and the subscript *i* means the *i*-th sample pair. We denote $|D^{t}|$ as the number of subset $D^{t}$, and $|C^{t}|$ as the number of classes contained in subset $D^{t}$. Only the subset $D^{t}$ is available and the labels of class $C^{t}$ are contained in $G^{t}$ during the *t*-th learning step, while the previously learned classes $C^{1:t-1}$ is regarded as the background class $C^{0}$. Due to the disjointness of classes contained in distinct subsets, then $C^{1:t-1}\cap C^{t}=\emptyset$. After the learning step *t*, the labels of $C^{t}$ are no longer available, and the classes $C^{t}$ in the *t*-th step are regarded as the background in the current subset $D^{t+1}$ during the learning step t+1. Therefore, learning new classes and maintaining the segmentation capacity of the old classes is a challenging research topic in recent years.

There are two main issues with the CSS task. On the one hand, if the old model is directly fine-tuned on the new data with the unavailable labels of the old class, the model will quickly forget the learned knowledge, which will lead to a sharp performance decline for the old tasks, i.e., catastrophic forgetting.

On the other hand, in the CSS task, the forthcoming classes are considered as the background in the old task, while when learning new classes, the old classes are considered as the background because the corresponding labels are not available. Therefore, the semantics of the background changes with the different learning steps—the so-called background semantic shift [12] problem. Once the model is supervised directly by the labels of data from the new step, the background semantic shift will constrain the model to reclassify the old classes as the background to aggravate catastrophic forgetting. The details of our method for alleviating the two problems are illustrated as follows:

### 3.2. Basic Framework

We first introduce the basic framework of continual semantic segmentation. We build our method on a fully convolutional neural network *M* consisting of a convolutional encoder Enc and a decoder, where the decoder consists of a decoding network Dec and an output layer *O*. In the learning step *t*, given an image $I^{t}$, we first input it into the encoder ${Enc}^{t}$ to get the deep feature map of the image $F^{t}\in R^{C\times \frac{H}{16}\times \frac{W}{16}}$, then feed it into the decoding network ${Dec}^{t}$ to get the decoded feature map $F_{o}^{t}$. Finally, the feature map $F_{o}^{t}\in R^{C_{o}\times \frac{H}{16}\times \frac{W}{16}}$ is fed into the output layer $O^{t}$ to get the final segmentation result $S^{t}\in R^{|C^{0:t}|\times H\times W}$. Our framework is formulated as:

(1) $$\begin{array}{cc} F^{t}={Enc}^{t} & \left(I^{t}\right);\;\;F_{o}^{t}={Dec}^{t}\left(F^{t}\right)\\ \; & S^{t}=O^{t}\left(F_{o}^{t}\right) \end{array}$$

In the new learning step t+1, we first initialize the current model $M^{t+1}$ using the old model $M^{t}$ obtained from the last training step *t*. To prevent the model from catastrophic forgetting during the learning process of new classes, we use the old model $M^{t}$ in the last step to perform knowledge distillation on the current model $M^{t+1}$ to maintain the knowledge of the old classes learned by the model and we perform novel knowledge distillation methods for the encoder and decoder of the model respectively to improve the stability of the model for old classes and the plasticity for new classes.

For the encoder, we focus on the comprehensive distillation of semantic information from multiple feature layers and propose the scale-hybrid group encoder semantic distillation (SGD). For the decoder, we propose a knowledge disentangling distillation (KDD) method that disentangles the old and new classes in the image and distills the old class features. In the following subsections, we will introduce our proposed distillation method in detail.

### 3.3. Scale-Hybrid Group Distillation

To mitigate catastrophic forgetting in continual semantic segmentation, most studies adopt the technique of knowledge distillation [7,8,10], which minimizes dissimilarities between features of the teacher and features of the student [59]. We denote the intermediate features of *l*-th layer in the encoder as $F_{l}$, the distillation loss between the old model and the current model defined by $L_{2}$ norm is as follows:

(2) $$\begin{array}{c} L_{l}^{dis}=\frac{1}{HW}\sum_{i=1}^{HW}{∥f_{l,i}^{t}-f_{l,i}^{t-1}∥}^{2} \end{array}$$

where $f_{l,i}^{t-1}$ are the features of $F_{l}$ at position *i* in the old model and $f_{l,i}^{t}$ are the features of the current model. However, simply applying vanilla $L_{2}$ norm to measure dissimilarities between features commonly can not achieve ideal performance, due to the lack of utilization of the richer semantic knowledge of the old model, including multi-scale object information, spatial position, and channel semantics. If we regularize $L_{l}^{dis}$ too much, it will constrain the features of the current model close to the old features, and damage the process of learning the new tasks. If we loosen $L_{l}^{dis}$ too much, it will make the model ignore the semantic knowledge of old tasks and lead to catastrophic forgetting. Therefore, adaptively distilling the important semantic features of the previous tasks and leaving more plasticity for the new tasks is vital for improving the performance.

**Figure 2:**
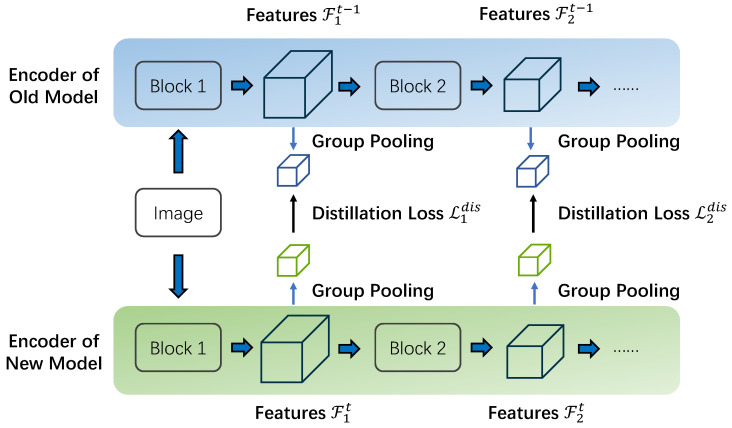
Illustration of scale-hybrid group distillation.

POD [43] distills pooled features at different layers between the old model and the new model to alleviate catastrophic forgetting. However, semantic segmentation requires more fine-grained information than the task of classification. We devise a novel Scale-hybrid Group Distillation method to distill features within each small cuboid of features, as shown in Figure 2. Specifically, we first divide features $F\in R^{C\times H\times W}$ into $G^{2}*K$ groups. For each group, the features can be represented as:

(3) $$\begin{array}{c} \begin{array}{cc} {\tilde{F}}_{i,j,k}=F & [\frac{kC}{K}:\frac{(k+1)C}{K},\frac{iH}{G}:\frac{(i+1)H}{G}\\ \; & \frac{jW}{G}:\frac{(j+1)W}{G}] \end{array} \end{array}$$

where i,j=1,…,G and k=1,⋯,K. Then, we apply the average pooling on each $f_{i,j,k}$:

(4) $$\begin{array}{c} {\bar{f}}_{i,j,k}=AvgPool\left({\tilde{F}}_{i,j,k}\right) \end{array}$$

We concatenate the pooled results of these cubes and obtain the final features for distillation:

(5) $$\begin{array}{c} \bar{f}=[{\bar{f}}_{1,1,1}∥,\cdots ∥,{\bar{f}}_{G,G,K}] \end{array}$$

The process of the proposed group-wise distillation measuring the dissimilarities by the pooled features is formulated as follows:

(6) $$\begin{array}{c} L_{l}^{dis}={∥{\bar{f}}_{l}^{t}-{\bar{f}}_{l}^{t-1}∥}^{2} \end{array}$$

To further keep the consistency of different scales between the old and current models, an average loss over multi-scale features of different layers is constructed:

(7) $$\begin{array}{c} L^{dis}=\frac{1}{L}\sum_{l=1}^{L}L_{l}^{dis} \end{array}$$

where *L* is the total amount of layers we distill. Through this scale-hybrid group distillation, we can effectively and comprehensively reserve the semantic knowledge of previous tasks and continually learn new tasks.

### 3.4. Knowledge Disentangling Distillation

In the process of distilling the features generated by the encoder, the feature map is considered as a whole, without distinguishing between different classes. However, an image often contains both new and old classes simultaneously and the background semantic shift problem makes the definition of background dynamically change over time, i.e., the foreground classes in the current task are regarded as the background in the old task. If the entire feature map is indiscriminately distilled using the old model, the semantic shift of the old model to the background will mislead the current model and limit the plasticity of the model. At the same time, the old model has a richer knowledge of identifying old classes and a stronger ability to represent the features of the old classes, which is also the most desired knowledge to be preserved during the distillation process. Therefore, in order to further explore the distillation of old classes by old models, we propose a knowledge disentangling distillation method to disentangle old and new classes and use the old model to distill the old class features more intently while reducing the interference in learning the new classes.

Specifically, our method disentangles the old and new classes and consists of three parts. First, we use the segmentation masks predicted by the old model in the last step as the pseudo-labels of the old classes. These pseudo-labels are used for capturing regions of the old classes and supervising the current segmentation results. Second, based on the spatial regions corresponding to the old classes in the pseudo-labels, the class-specific features are captured by the current model, and they are distilled by the old model. Finally, the ground truths of the current new classes are combined with the pseudo-labels of the old classes for the supervision of the output results of the current model.

#### 3.4.1. Pseudo-Label Generation

During different learning steps, old classes are annotated as background since their labels are not available. Therefore, the model cannot perceive the region corresponding to the old classes, and it is difficult to use the old model to distill the features of the old class regions generated by the current model. Moreover, the background semantic shift caused by the background semantics is constantly changing in different learning steps, which aggravates the catastrophic forgetting of the model.

To address this problem, since the old model has a stronger segmentation ability for the learned old classes, following Ref. [7], we leverage the old model to generate pseudo-labels for the old classes. In learning step *t*, given an image $I^{t}$ in the current dataset $D^{t}$, we first feed $I^{t}$ into the old model $M^{t-1}$ in the last step to generate segmentation predictions $S^{t-1}$ for the old classes. However, the segmentation mask $S^{t-1}$ of the old classes generated by the old model is not completely accurate. If the regions of old classes are captured completely according to $S^{t-1}$, many features of the non-old classes will also be included. To reduce these noises and interference, we use the median of the entropy of the old model’s prediction probabilities for each class in the current dataset as the threshold for determining the confidence of the model’s prediction. For position *i*, when the entropy of the probability of class *c* predicted by the old model is less than the threshold $\tau_{c}$, it means that the prediction confidence of position *i* is higher, and it is set as the foreground, otherwise the position is ignored. The updated pseudo-label ${\tilde{S}}^{t-1}$ is formulated as:

(8) $${\tilde{S}}_{i}^{t-1}=\left\{\begin{array}{ccc} S_{i}^{t-1}, & \; & if\;\;entropy\left(S_{i}^{t-1}\right)<\tau_{c}\\ \mu , & \; & otherwise \end{array}\right.$$

where μ denotes the position that is ignored and not calculated. Based on the obtained pseudo-labels ${\tilde{S}}^{t-1}$, we further perform the distillation of old class features and supervision of the segmentation results.

#### 3.4.2. Distillation of Old Class

Since the features of different classes are not distinguished, the incorrect recognition of the new classes by the old model will mislead the learning processing, and it also causes difficulties in distilling the knowledge of the old classes. To enhance the old classes distillation, after obtaining accurate old class pseudo-label ${\tilde{S}}^{t-1}$, we generate class region masks M corresponding to different old classes according to ${\tilde{S}}^{t-1}$, and distill the old class-specific features. In learning step *t*, given an image $I^{t}$, we first generate an output feature map $F_{o}^{t-1}$ and $F_{o}^{t}$ through the old and new models. For the old class *c*, we first obtain the area corresponding to *c* in the pseudo-label ${\tilde{S}}^{t-1}$, and get the area mask $M_{c}$ of class *c*.

Then we conduct element-wise multiplication of $M_{c}$ with $F_{o}^{t-1}$ and $F_{o}^{t}$ and use average pooling to fuse the features in the *c*-th class region to obtain the embedding features of class *c* generated by the old and new models. The equations are formulated as:

(9) $$\begin{array}{cc} f_{c}^{t-1} & =AvgPool(M_{c}\cdot F_{o}^{t-1})\\ f_{c}^{t} & =AvgPool(M_{c}\cdot F_{o}^{t}) \end{array}$$

Following the above approach, we disentangle the old and new classes and obtain the embedding features of the old classes generated by the old and the current model. Next, we perform distillation exclusively on old class features. Specifically, we constrain the embedding features of each old class generated by the current model to be similar to those generated by the old model, to more intently distill the old knowledge learned and reduce the interference of the current model to learn new classes. The equation for distillation loss is formulated as:

(10) $$L^{dist\_old}=\frac{1}{|C^{1:t-1}|}\sum_{c=1}^{|C^{1:t-1}|}\left|\right|f_{c}^{t}-f_{c}^{t-1}{\left|\right|}^{2}$$

#### 3.4.3. Learning of New Class

While maintaining the knowledge of old classes, the current models need to further learn new classes. To prevent catastrophic forgetting due to background semantic shift and supervise the predictions of new classes, we combine the pseudo-label of the old classes ${\tilde{S}}^{t-1}$ with the ground truth $G^{t}$ of the current new classes as the supervision of the current model. The combined label ${\hat{G}}^{t}$ is formulated as:

(11) $${\hat{G}}_{i}^{t}=\left\{\begin{array}{ccc} G_{i}^{t}, & \; & G_{i}^{t}\in C^{t}\\ {\tilde{S}}_{i}^{t-1}, & \; & {\tilde{S}}_{i}^{t-1}\in C^{1:t-1}\;and\;G_{i}^{t}=0\\ 0, & \; & otherwise \end{array}\right.$$

where we replace the background of $G^{t}$ with the pseudo-labels of the old classes of ${\tilde{S}}^{t-1}$.

We utilize the obtained combined labels for training to get the segmentation predictions generated by the current model. The segmentation loss is:

(12) $$L^{seg}=\frac{1}{HW}\sum_{i=1}^{HW}\sum_{c\in C^{0:t}}{\hat{G}}_{c,i}^{t}logS_{c,i}^{t}$$

## 4. Experiments

In this section, we evaluate the performance of our method on the Pascal VOC dataset [13] and ADE20K dataset [15].

### 4.1. Datasets, Protocols, and Metrics

#### 4.1.1. Datasets

The Pascal VOC dataset [13,60] is a mainstream benchmark dataset for object detection and semantic segmentation tasks in computer vision, which contains 20 different semantic classes with 10,582 images for training and 1449 images for testing.

The ADE20K dataset [15] is a large-scale scene understanding dataset, which contains 150 semantic classes, with 20,210 fully annotated images for the training process and 2000 fully annotated images for validation.

#### 4.1.2. Protocols

There are two different settings for continual learning for semantic segmentation: Overlapped and Disjoint. For overlapped setting, the data of each step further contains future classes, which is more consistent with realistic scenes. For disjoint setting, the data in each step only contains old classes $C^{0:t-1}$ learned in the previous steps and the current classes $C^{t}$, without the future classes, while old classes are labeled as the background.

Following existing methods [7,11], for Pascal VOC dataset [13], we evaluate our method on traditional continual semantic segmentation protocols, including VOC-19-1 (2 steps, first training on 19 classes, and then on 1 new class), VOC-15-5 (2 steps, first training on 15 classes, and then on 5 new classes), and VOC-15-1 (6 steps, first training on 15 classes, and then on 1 new class in each of the next 5 steps).

For ADE20K dataset [15], we evaluate on our method with similarly continual semantic segmentation protocols, including 100-50 (2 steps, first training on 100 classes, and then on 50 new classes), 50-50 (3 steps, first training on 50 classes, and then on 50 new classes in each of the next 2 steps) and 100-10 (6 steps, first training on 100 classes, and then on 10 new classes in each of the next 5 steps).

#### 4.1.3. Metrics

We use mean Intersection over Union (mIoU) as metrics to evaluate the performance of our method. This is a widely used evaluation metric in the field of semantic segmentation [1], which measures the accuracy of a segmentation algorithm by comparing the overlap between predicted and ground truth regions in an image. Specifically, we compute mIoU after the last step *T* for the initial classes $C^{1}$, for the incremented classes $C^{2}$, and for all classes $C^{T}$. These metrics represent the stability (the robustness to catastrophic forgetting), the plasticity (capacity to learn new classes), and the overall performance of the proposed model, respectively.

#### 4.1.4. Implementation Details

We utilized the Deeplab-V3 [18] architecture with ResNet-101 [61] as the backbone following current popular continual semantic segmentation methods [7,8,12]. The output stride of Deeplab-V3 is set to 16, and the in-place activated batch normalization [62] is applied in the backbone, which is pre-trained on the ImageNet [63].

We train the model on the Pascal VOC dataset for 30 epochs for every continual semantic segmentation step and the ADE20K dataset for 60 epochs for every continual semantic segmentation step, where the initial learning rate is $2\times 10^{-2}$ for the first step and $10^{-3}$ for all of the remaining steps and the batch size is set to 24 for both datasets. The learning rate is reduced exponentially with a decay rate of 0.9. We use the SGD optimizer with a momentum of 0.9 and weight decay rate of $10^{-4}$ during training. The feature maps used in our method are applied before ReLU with squared pixel values. During the process of continuous learning, we use the loss function proposed by MiB [12] following RCN [10]. Adhering to the definition of incremental learning, the task ID during inference is not accessible by the model, which needs to predict the target class in the set of all the seen classes.

### 4.2. Main Results

#### 4.2.1. Pascal VOC

As we can see in Table 1, we first compare our method with the current state-of-the-art continual semantic segmentation methods on the Pascal VOC dataset [13] under the overlapped settings. Our method is evaluated on different continual learning tasks, namely 19-1, 15-5, and 15-1, and shows consistent improvements over current popular methods. Compared with the most recent method GSC [64], our method achieves competitive performance under setting of 19-1, and outperforms it by 0.83% and 2.28% under settings of 15-5 and 15-1 for all classes respectively.

Under the disjoint settings, as shown in Table 2, our method still achieves significant performance improvements. Especially under the setting of long learning steps 15–1, our method outperforms GSC [64] by 1.58%, which has the best performance previously except for introducing additional data of RECALL [9]. Our method reduces the forgetting of old classes while improving the learning ability of new classes, and the model’s stability and plasticity are enhanced.

#### 4.2.2. ADE20K

In this part, we conduct experiments on the most challenging ADE20K dataset [15] for semantic segmentation to verify the effectiveness of the proposed method. The quantitative results are shown in Table 3. Our method is evaluated on multiple continual learning tasks, i.e., 100-50, 100-10, and 50-50, and it outperforms the current popular continual semantic segmentation methods.

ILT [6] builds a bridge between continual learning and semantic segmentation with weak effectiveness. Our method outperforms it by a large margin of 18.72%, 23.52%, and 34.05% under the 100-50, 100-10, and 50-50 settings, respectively. MiB [12] aims to address the background class shift problem, while our method proposes knowledge disentangling distillation to achieve higher performance, i.e., our method outperforms it by 2.93%, 3.91%, and 5.9% under settings of 100-50, 100-10, and 50-50, respectively. PLOP [7] extracts features in intermediate layers to make knowledge distillation in order to alleviate the issue of catastrophic forgetting, while our method proposes scale-hybrid group distillation to obtain greater improvement, e.g., our method outperforms it by 2.78%, 2.82%, and 3.55% under settings of 100-50, 100-10, and 50-50, respectively. Architectural-based RCN [10] designs two parallel network branches for storing previous information and learning new categories to avoid catastrophic forgetting, while our method obtains greater improvement, e.g., our method outperforms it by 1.22%, 0.72%, and 3.04% under settings of 100-50, 100-10, and 50-50, respectively. In addition, because the proposed method can retain most of the information from previous tasks, it has achieved greater improvement in more challenging experimental settings (6 steps) and obtains a 1.87% improvement compared with the second place overall.

### 4.3. Ablation Study

In this section, we conduct a set of ablation experiments to analyze the effectiveness of the different components of our proposed method.

#### 4.3.1. Distillation Mechanism

We conduct ablation experiments under the overlapped setting of 15-1 on the Pascal VOC dataset. As shown in Table 4, the different components of our method achieve considerable improvements. PLOP [7] extracts features in intermediate layers, and RCN [10] designs two parallel network branches for storing previous information and learning new categories to alleviate the issue of catastrophic forgetting caused by knowledge distillation, respectively. The proposed scale-hybrid group distillation (SGD) brings performance improvement for both PLOP and RCN. At the same time, knowledge disentangling distillation (KDD) obtains consistent improvements both in combination with PLOP and RCN. In our method, both SGD and KDD have their own improvements. When combining SGD and KDD, it achieves the state-of-the-art performance of 63.08%, which demonstrates the effectiveness of the proposed method.

#### 4.3.2. Different Group Pooling Kernel Sizes

In this part, we conduct ablation experiments under the overlapped setting of 15-1 on the Pascal VOC dataset. As shown in Table 5, the different group pooling kernel sizes play an important role in the proposed scale-hybrid group distillation (SGD) method. In practice, we set the group pooling kernel as a cube and divide the intermediate feature maps of ResNet [61] into three stages (C2C3, C4, C5). The performances of five different kernel sizes combinations of the three stages are compared. From the Table, the kernel size (8,8,8) achieves the worst performance, with one of the reasons being that the huge kernel size leads the network to ignore some detailed information. With the kernel size decreasing, e.g., (8,8,8) to (4,4,4), (4,4,4) to (2,2,2), the performance becomes better, where the kernel size (2,2,2) achieves a performance of 63.02% and outperforms the kernel size (8,8,8) by 13.12% mIoU. In addition, we conduct the experiments with mixed kernel size combinations, e.g., (2,4,8) and (8,4,2). Finally, the kernel size (8,4,2) achieves the best performance of 63.08%.

#### 4.3.3. Different Pooling Methods

In this part, we conduct ablation experiments under the overlapped setting of 15-1 on the Pascal VOC dataset. As shown in Table 6, we compared five pooling methods of knowledge distillation. It can be observed that the proposed group pooling method preserves more semantic information compared with other pooling methods and achieves the best performance.

### 4.4. Visualization

**Figure 3:**
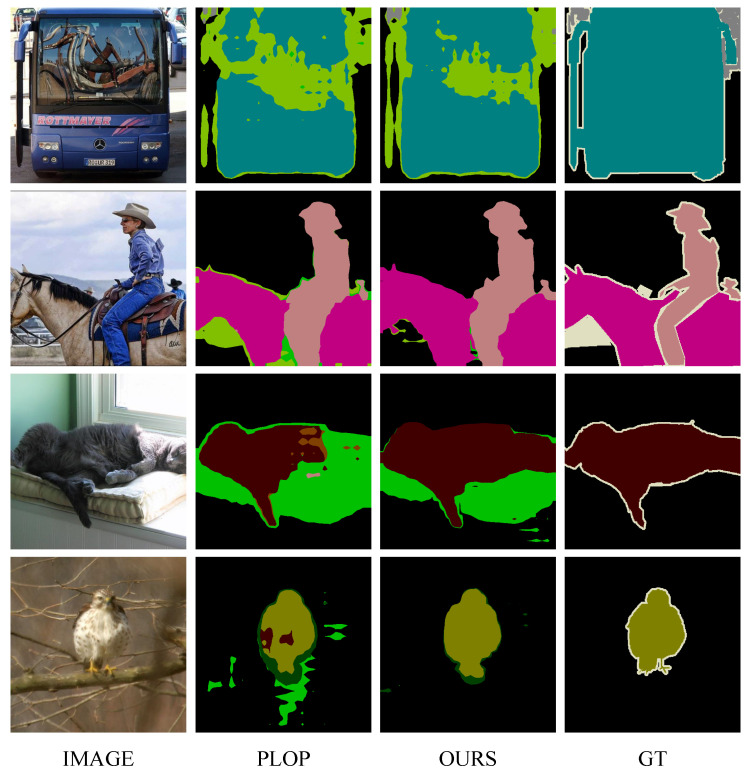
Visualization of PLOP [7] and our predictions on Pascal VOC dataset [13]. ‘Image’ denotes the original image, and ‘GT’ denotes the ground truth.

**Figure 4:**
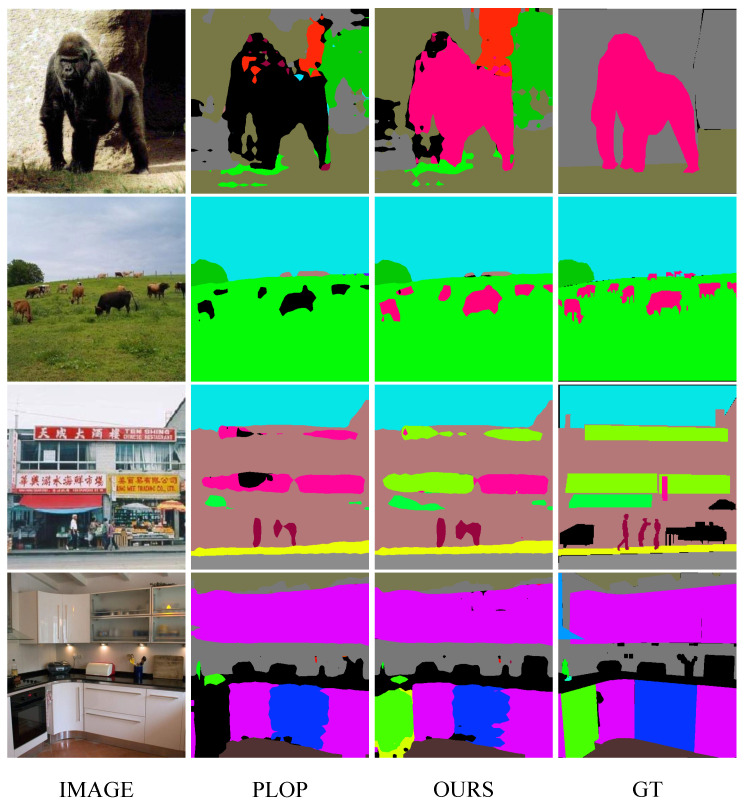
Visualization of PLOP [7] and our predictions on the ADE20K dataset [15]. ‘Image’ denotes the original image, and ‘GT’ denotes the ground truth.

**Figure 5:**
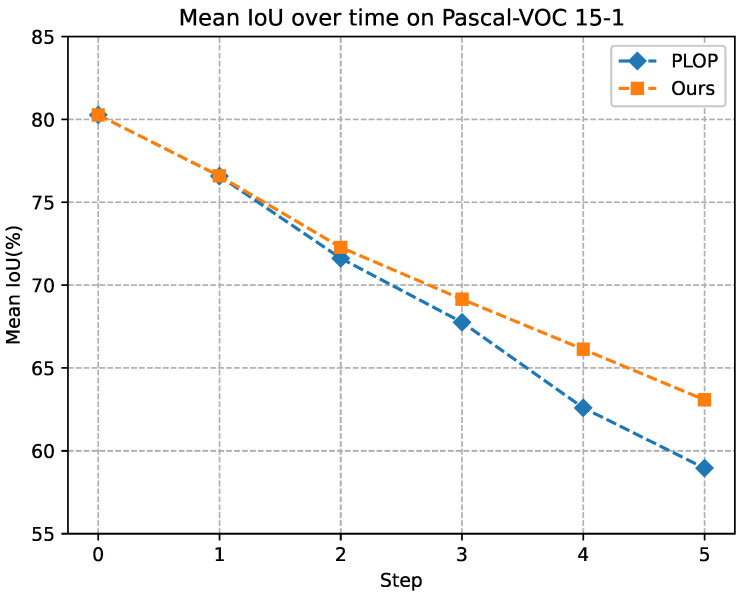
mIoU evaluation under the overlapped setting of 15-1 on the Pascal VOC dataset.

**Figure 6:**
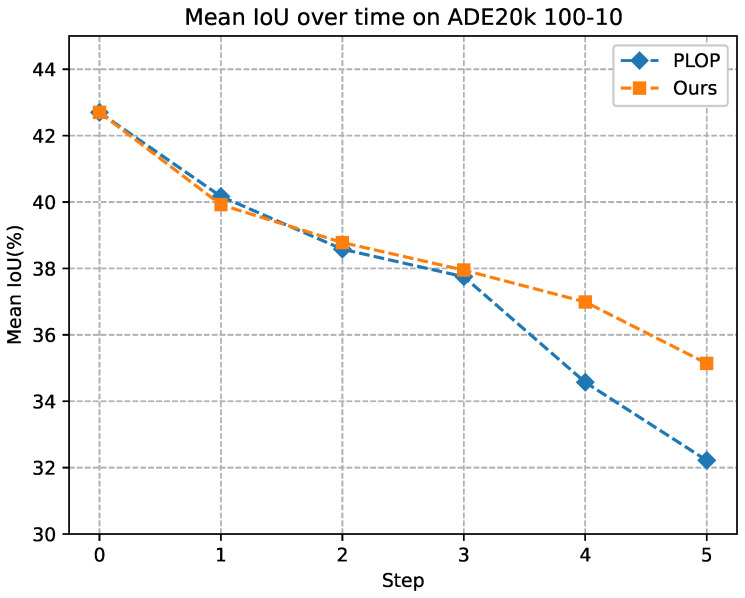
mIoU evaluation under the overlapped setting of 100-10 on ADE20K dataset.

**Figure 7:**
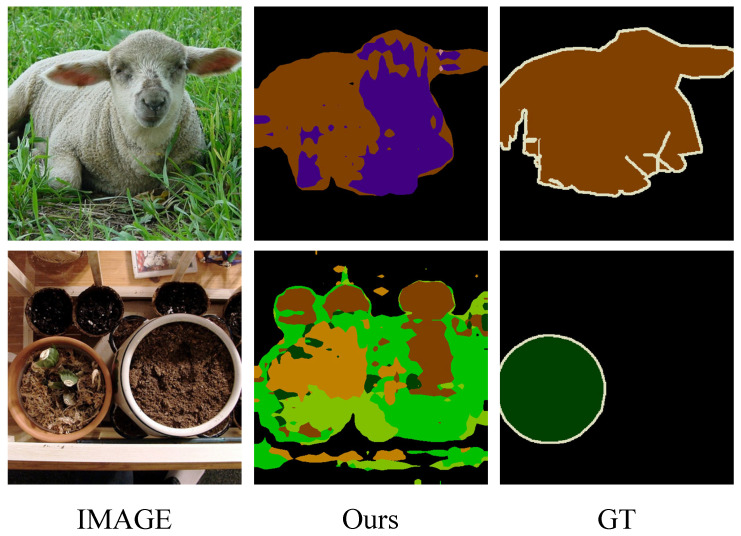
Visualization of two typical failure examples. ‘Image’ denotes the original image, and ‘GT’ denotes the ground truth.

In this section, we provide several visualization examples of the typical PLOP [7] and our method for qualitative comparison, as shown in Figure 3 and Figure 4. The visualization results demonstrate that the proposed method is significantly improved compared with PLOP. Some old categories are forgotten and not segmented well by PLOP, but our method makes an accurate segmentation. Besides, regarding the results shown in Figure 5 and Figure 6, while mIoU for PLOP deteriorates after only a handful of steps, our method’s mIoU remains very high throughout, indicating improved resilience to catastrophic forgetting and background semantic shift. In addition, we present two typical failure examples in Figure 7. The first row represents the problem of category confusion caused by catastrophic forgetting of the old categories, while the second row represents the challenge of segmentation for hard samples of new categories due to background semantic shift. In future work, we will employ more powerful feature extraction networks such as a transformer to further address these challenges.

## 5. Conclusions

In this paper, we propose a novel continual semantic segmentation method that utilizes two key components: the scale-hybrid group distillation for the encoder and the knowledge disentangling distillation for the decoder. Our scale-hybrid group distillation facilitates knowledge transfer from the feature encoder of the old to the new model at different scales through group pooling refinement so that the new model can preserve the old model’s abundant semantic information. Moreover, the knowledge disentangling distillation prevents the semantic shift of the background by focusing on the regions of old classes and reducing incorrect guidance from the old model. We evaluated our method on two challenging continual semantic segmentation datasets: the Pascal VOC and ADE20K datasets. The results demonstrate the effectiveness of our method and its superiority over other state-of-the-art methods.

## Figures and Tables

**Table 1:** Continual semantic segmentation results under the *Overlapped* settings of VOC-19-1, VOC-15-5, and VOC-15-1 benchmarks. Best in **bold**.

Method	19-1 (2 Steps)			15-5 (2 Steps)			15-1 (6 Steps)		
	0–19	20	*All*	0–15	16–20	*All*	0–15	16–20	*All*
ILT [6]	67.75	10.88	65.05	67.08	39.23	60.45	8.75	7.99	8.56
MiB [12]	71.43	23.59	69.15	76.37	49.97	70.08	34.22	13.50	29.29
SDR [8]	69.10	32.60	67.40	75.40	52.60	69.90	44.70	21.80	39.20
PLOP [7]	75.35	37.35	73.54	75.73	51.71	70.09	65.12	21.11	54.64
RECALL [9]	67.90	53.50	68.40	66.60	50.90	64.00	65.70	47.80	62.70
UCD [65]	71.40	47.30	70.00	77.50	53.10	71.30	49.00	19.50	41.90
CAF [66]	75.50	34.80	73.40	77.20	49.90	70.40	55.70	14.10	45.30
RCN [10]	-	-	-	78.80	52.00	72.40	70.60	23.70	59.40
RBC [11]	**77.26**	**55.60**	**76.23**	76.59	52.78	70.92	69.54	**38.44**	62.14
SPPFA [67]	76.50	36.20	74.60	78.10	52.90	72.10	66.20	23.30	56.00
AWT [68]	-	-	-	77.30	52.90	71.50	59.10	17.20	49.10
GSC [64]	76.90	42.70	75.30	78.30	54.20	72.60	72.10	24.40	60.80
Ours	77.01	39.97	75.25	**78.82**	**56.16**	**73.43**	**73.92**	28.37	**63.08**
Joint	77.40	78.00	77.40	79.10	72.56	77.39	79.10	72.56	77.39

**Table 2:** Continual semantic segmentation results under the *Disjoint* settings of VOC-19-1, VOC-15-5, and VOC-15-1 benchmarks. Best in **bold**.

Method	19-1 (2 Steps)			15-5 (2 Steps)			15-1 (6 Steps)		
	0–19	20	*All*	0–15	16–20	*All*	0–15	16–20	*All*
ILT [6]	69.10	16.40	66.40	63.20	39.50	57.30	3.70	5.70	4.20
MiB [12]	69.60	25.60	67.40	71.80	43.30	64.70	46.20	12.90	37.90
SDR [8]	69.90	37.30	68.40	73.50	47.30	67.20	59.20	12.90	48.10
PLOP [7]	75.37	38.89	73.64	71.00	42.82	64.29	57.86	13.67	46.48
RECALL [9]	65.20	**50.10**	65.80	66.30	49.80	63.50	66.00	**44.90**	**62.10**
UCD [65]	73.40	33.70	71.50	71.90	49.50	66.20	53.10	13.00	42.90
CAF [66]	75.50	30.80	73.30	72.90	42.10	65.20	57.20	15.50	46.70
RCN [10]	-	-	-	75.00	42.80	67.30	66.10	18.20	54.70
RBC [11]	76.43	45.79	75.01	75.12	**49.71**	**69.89**	61.68	19.52	51.60
SPPFA [67]	75.50	38.00	73.70	75.30	48.70	69.00	59.60	15.60	49.10
GSC [64]	75.90	31.00	74.00	74.40	45.80	67.60	67.20	19.20	55.80
Ours	**77.10**	39.91	**75.33**	**75.42**	44.84	68.14	**69.38**	19.00	57.38
Joint	77.40	78.00	77.40	79.10	72.56	77.39	79.10	72.56	77.39

**Table 3:** Continual semantic segmentation results under the *Overlapped* settings of ADE-100-50, ADE50-50, and ADE-100-10 benchmarks. Best in **bold**.

Method	100-50 (2 Steps)			50-50 (3 Steps)			100-10 (6 Steps)		
	0–100	101–150	*All*	0–50	51–150	*All*	0–100	101–150	*All*
ILT [6]	18.29	14.40	17.00	3.53	12.85	9.70	0.11	3.06	1.09
MiB [12]	40.52	17.17	32.79	45.57	21.01	29.31	38.21	11.12	29.24
PLOP [7]	41.87	14.89	32.94	48.83	20.99	30.40	40.48	13.61	31.59
UCD [65]	42.12	15.84	33.31	47.12	24.12	31.79	40.80	15.23	32.29
RCN [10]	42.30	18.80	34.50	48.30	25.00	32.50	39.30	17.60	32.10
RBC [11]	**42.90**	21.49	**35.81**	49.59	26.32	**34.18**	39.01	**21.67**	33.27
SPPFA [67]	42.90	19.90	35.20	**49.80**	23.90	32.50	41.00	12.50	31.50
AWT [68]	40.90	24.70	35.60	46.60	**26.85**	33.50	39.10	21.28	33.20
GSC [64]	42.40	19.20	34.80	46.20	26.20	33.00	40.80	17.60	32.60
Ours	42.32	**22.38**	35.72	48.71	25.18	33.22	**43.23**	20.83	**35.14**
Joint	43.90	27.20	38.30	50.90	32.10	38.30	43.90	27.20	38.30

**Table 4:** The final mIoU (%) of the ablation study of our method. SGD denotes Scale-hybrid Group Distillation and KDD denotes Knowledge Disentangling Distillation. All experiments are conducted under the overlapped setting of 15-1 on Pascal VOC dataset.

PLOP [7]	RCN [10]	SGD	KDD	15-1 (6 Steps)
✔				58.32
	✔			59.64
		✔		62.03
✔			✔	62.17
	✔		✔	62.81
		✔	✔	63.08

**Table 5:** The final mIoU (%) of the ablation study about different group pooling kernel sizes. The numbers in brackets denote the pooling kernel sizes of three stages in sequence. All experiments are conducted under the overlapped setting of 15-1 on Pascal VOC dataset.

Kernel Sizes	15-1 (6 Steps)
(8,8,8)	49.90
(4,4,4)	58.96
(2,2,2)	63.02
(2,4,8)	49.97
(8,4,2)	63.08

**Table 6:** The final mIoU (%) of the ablation study about different pooling methods. All experiments are conducted under the overlapped setting of 15-1 on Pascal VOC dataset.

Method	15-1 (6 Steps)
Strip Pooling	62.17
Spatial Pooling	48.55
Channel Pooling	44.12
Spatial and Channel Pooling	62.81
Max Pooling	62.42
Group Pooling	63.08

## Data Availability

Publicly available datasets were analyzed in this study. The Pascal VOC dataset can be found here (accessed on 1 October 2022): http://host.robots.ox.ac.uk/pascal/VOC/. The ADE20K dataset can be found here (accessed on 1 October 2022): https://groups.csail.mit.edu/vision/datasets/ADE20K/.
